# Supplementary figures and images for: Difficulty and pleasure in the comprehension of verb-based metaphor sentences: A behavioral study
Source: PLoS One. 2022 Feb 11;17(2):e0263781. doi: 10.1371/journal.pone.0263781 (PMC8836342; doi:10.1371/journal.pone.0263781)

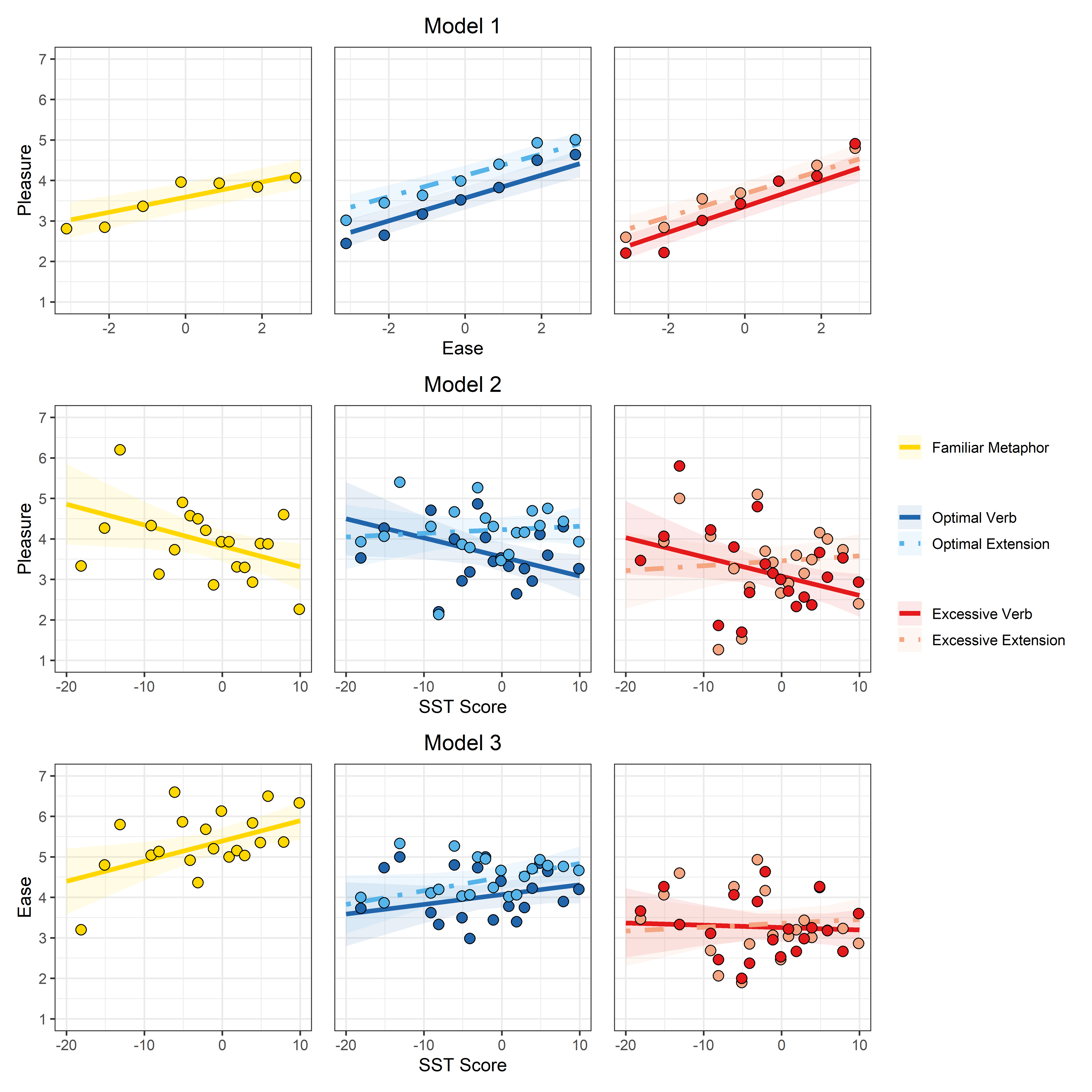

Supplement: S1 Fig — The key observation is that none of the panels suggest a U-shape in the behavioural data and the linear models appear to fit the data reasonably well. Left column shows results for familiar metaphors, middle column shows results for optimal verb and optimal extension conditions, right column shows results for excessive verb and excessive extension condition. Top row: relationship between ease of comprehension and pleasure (Model 1). Middle row: relationship between SST Score (semantic knowledge) and pleasure (Model 2). Bottom row: relationship between SST Score (semantic knowledge) and ease of comprehension (Model 3). (TIF) [file pone.0263781.s001.tif]
