# Supplementary material for: Difficulty and pleasure in the comprehension of verb-based metaphor sentences: A behavioral study
Source: PLoS One. 2022 Feb 11;17(2):e0263781. doi: 10.1371/journal.pone.0263781 (PMC8836342; doi:10.1371/journal.pone.0263781)
Supplement: Attachment — Submitted filename: Response to Reviewers.docx [file pone.0263781.s002.docx]

**Response to Reviewers (‘Difficulty and pleasure in the comprehension of verb-based metaphor sentences: a behavioral study’)**

Dear editors and reviewers,

We greatly appreciate the consideration given to this manuscript, and the helpful questions, recommendations, and critiques. We have sought to address each of the points raised in the manuscript. Below, we have reproduced the editor’s and reviewer’s comments followed by our replies (in bold), indicating, where appropriate, the manuscript line numbers that correspond with our changes and corrections.

If you or anyone else has any questions regarding these changes, or would like to suggest other amendments, please feel free to get in touch.

With thanks for your time and consideration,

Sincerely,

Patrick Errington (corresponding author)

Daniel Mirman

Melissa Thye

# Academic Editor Comments

1) the logic underlying the use of your individual difference variable (SST) needs to be clarified (see Reviewer 1), as well as the appropriateness of your analyses for examining its role (see Reviewer 2).

**We thank the editor and both reviewers for the suggestion to further clarify the Semantic Similarities Test and its analysis. We have explained the importance of the SST as a means of ascertaining the effects of an individual’s capacity for metaphorical thinking, which we hypothesised would impact the ease with which a given individual would be able to resolve all the variations of metaphors with which they were presented. This was explained with regard to Reviewer 1’s comments (ll. 224–233). This hypothesis was borne out in our study, which found those with higher SST scores to have greater ease in comprehending all categories of metaphor; surprisingly, however, those with higher SST scores did not show greater pleasure in comprehending the ‘optimal extension’ condition, nor did they show an increase in pleasure in comprehending the ‘excessive extension’, which we would have expected if their increased metaphor comprehension capacities had only moved the apex of the inverted U-shaped relationship toward the more challenging phrases. This effect is addressed in our discussion (ll. 693–707)**

2) as noted by Reviewer 1, there is a substantial literature on the relationship between aesthetic preferences and processing fluency/difficulty that is not referenced in your paper. You should include some of this literature in your revision; doing so may help you clarify the meaning of your results.

**We greatly appreciate reviewer 1’s suggested literature regarding aesthetic preference and processing fluency, and we found it quite helpful in framing our study (see additions ll. 113­–132).**

3) some of the analyses you report may be less than optimal for the issues you are investigating (see especially Reviewer 2). As noted by Reviewer 2, examining the ease-pleasure relationship separately for the different categories may result in the hypothesized curvilinear relationship being obscured.  Why not examine the ease-pleasure relationship across the entire data set? As well, there appears to be some redundancy in some of the models you test because you simultaneously include comprehension ease as well as metaphor category (which is based, in part, on comprehension ease).

**We appreciate the recommendation for a simpler analysis approach, which we have added to the manuscript. A more detailed description is included below in our response to the reviewer’s comment. We have also retained our original analyses because we believe they provide useful additional insight into the results.**

4) you should describe how your sample size was determined and address the issue of power to detect your predicted effects.

**We have clarified (ll. 458–463) that due to the novelty of this stimulus set and our experiment design, there was not a strong basis for predicting an effect size, which is necessary for a proper power analysis. Instead, we determined the sample size based on sample sizes from prior metaphor comprehension studies and practical limitations. We hope that, by making available our full stimulus set and analysis code, this study will provide the basis for future power calculations.**

5) participants in your experiment provided four ratings.  However, the analyses you report include only two (pleasure and ease).  I assume you’ve undertaken analyses with the other two rating scales (imageability and emotion) and some mention should be made of them (at the very least in a footnote).

**Those other two ratings (imageability and emotion) were included for later exploratory analyses, which have not been conducted yet. Because we did not have clear hypotheses about them and they are not directly related to the hypotheses evaluated here, we feel that they are outside the scope of this manuscript and have not added them. We only mention that those ratings were collected in the Methods for the purpose of transparency.**

# Reviewer 1

1. It is difficult to determine what kind of “pleasure” the authors are trying to address. The terminology varies: striking, thrilling, affective, pleasurable, etc. There is a substantial psychometric issue lurking here that should be addressed more directly. The authors may find it useful to locate “effective, satisfying, or powerful” (the wording eventually chosen for their ratings; pp. 18-19) within the most comprehensive available survey of aesthetic “emotions” (Schindler et al. 2017). Although the author’s formalist intent is explicit (“how much you liked the way the message was expressed”; l. 434), the kind of “affectiveness” that is at stake also remains obscure.
Schindler, I., Hosoya, G., Menninghaus, W., Beermann, U., Wagner, V., Eid, M., & Scherer, K. R. (2017). Measuring aesthetic emotions: A review of the literature and a new assessment tool. PLOS ONE, 12(6), e0178899. <https://doi.org/10.1371/journal.pone.017889>

**This point is well noted, and we thank the reviewer for these recommendations. We have sought to clarify (ll. 509–517) that the increase in aesthetic response (which we have, perhaps overly simply, termed ‘pleasure’) is more precisely an increase of affect in a broad sense rather than one or another particular ‘aesthetic emotion’ like those surveyed by Schindler et al. In our case, we sought to test whether whatever ‘content’ feeling was elicited by a familiar sentence could be increased or intensified by the variations.**

2. The authors indicate that their research is “informed” by Giora’s “optimal innovation hypothesis” (see especially, Giora et al. 2017).
a) One issue in Giora’s work is how to differentiate default literal, default metaphoric, and non-default metaphoric sentences so that metaphoric default salience can be assessed independently of metaphoric non-default salience. It is somewhat disconcerting, then, to learn that the authors did not include the default literal sentences in Study 1 because “we were only interested in metaphor comprehension” (l. 368). Giora is primarily interested in metaphor comprehension, too; so, the reason for ignoring differences between default literal and default metaphoric sentences should be more clearly articulated—and in terms she would understand.

**We again thank the reviewer for their probing questions, and we have sought to explain (ll. 429–439) that, where for Giora et al., the ‘default’ meaning is primarily non-metaphoric with a variation that introduces a metaphoric/figurative component (one of her examples is ‘A piece of paper’ which becomes ‘A *peace* of paper’), in our case, the ‘default’ meaning from the phrase was purely metaphoric. In ‘I run for office’, there is (according to studies and surveyed in Holyoak and Stamenkovic 2017) little recourse to any literal running, whereas we hypothesise that in ‘I dash for office’ there may be a dual activation of both the metaphorical meaning (‘I seek an elected office’) and some aspect of a literal meaning (‘I run quickly to achieve something physical). In this case the familiar metaphors provide an optimal control condition because they can be matched to the variations in terms of general meaning, figurativeness, and basic psycholinguistic properties (length, word frequency, etc.).**

b) A related but separate issue is how to differentiate metaphoric sentences so that, independently of defaultness, metaphoric vehicles can be contrasted according to the extent to which they are “domain-specific” (l. 167). The authors hypothesize that excessive domain specificity may make a metaphor too difficult to be pleasurable. However, emphasizing domain-specificity may oversimplify the problem. For example, Katz and Al-Azary (2017) differentiate (a) the distance between the domain of the topic and the domain of the vehicle; (b) the semantic density of the topic and of the vehicle; and (c) the specificity of the topic or vehicle within their respective domains (“domain-specificity”?). The authors should be encouraged to compare the Katz and Al-Zahry framework with their own. For example, perhaps they can clarify whether their use of word2vec (l. 267) converges with the Katz and Al-Zahry version of computational semantics.
Katz, A. N., & Al-Azary, H. (2017). Principles that promote bidirectionality in verbal metaphor. Poetics Today, 38(1), 35–59. <https://doi.org/10.1215/03335372-3716215>

**The reviewer’s suggestion to relate our work to that of Katz and Al-Azary is much appreciated and we have expanded our discussion regarding ‘domain-specificity’ to incorporate some of the ‘semantic density’ language offered by Katz and Al-Azary (ll. 199–208). We have specified that the word2vec norming does indeed converge with their computational methods for determining that density.** **This is also noted in the ‘Objective Measures’ subsection of the ‘Stimulus Development’ section (ll. 321–322).**

c) Contextualizing their hypothesis within the Katz and Alzary (2017) framework may also enable the authors to clarify their rationale for including the Semantic Similarities Test (SST) as an individual differences measure of “the ability to identify conceptual mappings between words” (l. 181). The SST assesses “crystalized verbal intelligence” by evaluating a person’s ability to find similarities between two concepts. Was this measure expected to reflect (a) the capacity to identify similarities between vehicle and topic concepts even when they are “domain-specific”; (b) the capacity to identify similarities between vehicle and topic concepts even when they are from distant domains; or (c) the capacity to identify similarities between vehicle and topic concepts because they are (especially for some individuals) semantically dense? As it stands, the SST is not conceptually well coordinated with the author’s research paradigm.

**Further discussion of the Semantic Similarities Test and its hypothesised and tested role in affecting metaphor phrase processing ease/difficulty has been incorporated (ll. 224–233), building upon discussion of Katz and Al-Alzary’s frameworks (see point above).**

d) It is a bit unsettling that there is no reference to other literature indicating that aesthetic preferences are determined by processing fluency/difficulty (cf. Reber et al., 2004), including the tradition of proposed curvilinear relations between object complexity and interest/pleasure (e.g., Berlyne, 1971). Research in the latter tradition substantiates how difficult it is to assess curvilinear relations because the direction of the relationship differs at different levels of the variables. More to the point, it is not clear where to locate “optimal” and “excessive” metaphors on the hypothesized curvilinear relation with pleasure. That difficulty should at least be mentioned.

**The reviewer’s suggestion to relate our work more explicitly to the history of proposed curvilinear relations between complexity and interest/pleasure is greatly appreciated. An expanded discussion of this history (ll. 113–132) has been included, leading to further discussions of the SST (ll. 216–233), in which we reference how our hypotheses contradict the predominantly linear hypotheses regarding processing fluency (e.g., Reber et al., 2004), wherein the difficulties in locating our stimulus categories along the hypothesized inverted U-shape relationship are indicated in response to a previous suggestion.**

3. Perhaps the most innovative aspect of the authors’ design is their attempt to examine novel extensions of familiar metaphors. In this reviewer’s judgment, their procedures for doing so are promising. The procedures used to develop these extended metaphors led to at least two interesting results. First, optimal extensions and excessive extensions were rated as the “most figurative” (l. 358). Second, metaphor extensions were rated as more pleasurable than verb variants, indicating that “innovations that increase … richness can increase pleasure without decreasing difficulty. These results are worth building on in future research efforts.

**We thank the reviewer for this positive appraisal of our procedures and results, and their encouragement for future research efforts.**

a) To explain these results, the authors emphasize “textual and situational context.” However, it may be more promising to examine specifically extended metaphors. The authors offer the following hypothesis: “The figurative meaning of the familiar metaphor is likely to be already active when the reader reaches the extension, which can more easily create the pleasurable tension between default and non-default interpretations.” The possibility of examining the interplay (and tension) between a familiar metaphor and a subsequent extension is within these authors’ methodological reach.

b) As their project unfolds, the authors may want to take advantage of Sullivan’s (2019) recent examination of mixed metaphors, some of which are subject to the domain specificity problem that makes them anathema in scholarly circles—but perhaps of particular interest to the authors.
Sullivan, K. (2018). Mixed metaphors: Their use and abuse. Bloomsbury Publishing.

**We wish again to express our thanks the reviewer for these two thoughtful suggestions for future areas of investigation.**

4. A not-so-important note (l. 290): a 7-point rating scale is not a “Likert scale.” This common misuse of that phrase should not be repeated here.

**We have removed the term ‘Likert’ as requested.**

# Reviewer 2

In this paper, the authors examine the “optimal innovation hypothesis”, which posits that language is most enjoyable when it evokes a non-default response, but also brings to mind the default response so that the two responses can be weighted against one another. The authors examined this hypothesis by carefully constructing a stimulus set of metaphors and obtaining subjective ratings of pleasurability for these metaphors.

First, the paper was well-written. It discussed an interesting theory within figurative language research. Also, I really enjoyed the writing style. I thought the examples used were interesting and made the paper exciting, and the authors also explained the theories and hypotheses clearly.

Second, I think the stimuli for this study are very nice, and I appreciate that the authors made them available on OSF. The authors carefully considered a variety of factors, including semantic distance measures and frequency. I also totally agree with the authors that there is an overemphasis on examining nominal metaphors, and I appreciate that they developed a set of verb metaphors and metaphor extensions and made these publicly available. I was very impressed by this aspect of the study.

That being said, I have major concern with the analyses the authors conducted to examine their hypotheses (Section 3.2: Results and Discussion). I question the logic of these analyses for a couple reasons.

1) First of all, the authors mention that there was no U-shaped curve observed in the data. However, to examine this, they split the familiar, optimal, and excessive metaphors into separate categories and examined these curves separately (Supplementary Figure). I think this defeats the purpose as this restricts the range of the data in each case. The whole point of the optimal and excessive categories was that they were supposed to increase difficulty, so it doesn’t make sense to me why difficulty would be examined within each of these categories separately. Wouldn’t it make more sense to either 1) look at the relationship between ease and pleasure collapsed across all the metaphors, or 2) compare the means between the categories directly (metaphors in the optimal category should have significantly higher pleasurability ratings than the familiar and excessive categories)? This criticism carries through to the first lme model (Model 1). Both the categories and the continuous ratings of ease were included in the model, but aren’t these essentially the same variable? Therefore, (if I understand lme correctly) if there is a significant effect of a category, this effect is independent of ease since ease is a variable in the model. However, the categories were specifically constructed to vary in ease, so the critical variable in the category is being controlled out of the category. Also, I didn’t quite get the logic of Model 2. It considered individual differences, but the relative differences of ease between the metaphors should still be preserved regardless of individual differences in figurative comprehension, so I’m also not sure how that tests the hypothesis.

   In the discussion, the authors state “the optimal metaphor extensions were rated as intermediate in terms of ease of comprehension, but highest in terms of pleasure”. And also, “familiar metaphors were rated both easier and more pleasurable than ‘optimal’ verb variants, which were rated both easier and more pleasurable than ‘excessive’ verb variants”. But these comparisons were not tested statistically, and weren’t really discussed much in the Results. I think the lme models obscure these findings, which are probably the most interesting part of the data.

I think this is a fixable problem though. I would recommend just conducting some standard statistics on the data, namely, an ANOVA between the categories (not considering ease ratings) and a correlation between ease and pleasurability (independent of the categories). It might be beneficial to run some separate ANOVAs to compare both optimal vs. excessive, but also verb vs. extension, since there seemed to be a trend in extensions being more pleasurable. I think this would more directly test your hypotheses and would make the Results section better fit with the Discussion section.

**We thank the reviewer for suggesting a simpler and more straightforward analysis strategy. Looking at the ease-pleasure relationship collapsed across qualitatively different types of metaphors would create the possibility of Simpson’s Paradox, so we have followed the second suggestion and now include simpler analyses of (i) Ease differences between conditions (to validate the experimental manipulation) and (ii) Pleasure differences between conditions (to test the critical hypotheses). We kept the analyses within the LME framework, though these simpler analyses are analogous to a repeated-measures ANOVA (LME allows more flexible specification of crossed random effects of participants and items). These analyses show monotonic differences in ease (Familiar > Optimal > Excessive) for both verb and extension manipulations. For pleasure ratings, there is a U-shape for extensions, but not for verbs.**

**Although the metaphor types differ in average ease of comprehension, there is substantial overlap and the range of ease values is very similar across the different types. So in addition to the simpler model suggested by the reviewer, we tested the more complex model that includes both variables (ease and metaphor type) – it allows testing whether the ease-pleasure relationship holds within categories and differs between categories.**

As one additional small point, I didn’t understand the NGram frequency graph on page 14. I think the axes need to be labelled or else the graph needs to be better explained in the caption.

**Thank for bringing this to our attention. We have elaborated the explanation in the Figure 1 caption.**

Overall, I think this paper has a lot of potential. The stimuli were very carefully constructed, and the data seems quite interesting. However, in my opinion, I think the analyses were somewhat inappropriate for the reasons stated above.
