# Supplementary material for: Difficulty and pleasure in the comprehension of verb-based metaphor sentences: A behavioral study
Source: PLoS One. 2022 Feb 11;17(2):e0263781. doi: 10.1371/journal.pone.0263781 (PMC8836342; doi:10.1371/journal.pone.0263781)
Supplement: Attachment — Submitted filename: Response to Reviewers2.docx [file pone.0263781.s003.docx]

Response to Reviewers

Dear Thomas Holtgraves,

Thank you very much for your continued scrutiny of our manuscript and for sending along our reviewers’ suggestions for further refinement.

We have made several alterations to the sections that they indicated, most notably adding a discussion of the potential for refinement of our terms ‘pleasure’ or ‘affectiveness’ (**l. 747–765**) and further clarifications to the potential role of semantic neighborhood density (SND) in this and future studies (**l. 245–260 and 688–707**), while removing an unclear point regarding our use of word2vec (**l. 320–322**). (A more detailed response to each point raised by the reviewers is included below.) We believe these amendments add considerable clarity to the manuscript and its place in metaphor scholarship, and we hope that it will satisfy both reviewers.

We all deeply appreciate both reviewers’ continued consideration and advice regarding this manuscript. Their work has provided us with a great many new ideas and has proven invaluable not only for this article but for our further research projects more broadly.

If it is possible to pass along our gratitude to them, we would greatly appreciate it.

With renewed thanks and with all best wishes,

Dr Patrick Errington (corresponding author), Dr Dan Mirman, and Melissa Thye

Reviewer #1: I indicated that all of the present reviewer's comments have been addressed, but I still have qualms about how effectively two of those comments were addressed. First, although it is not possible to undo the difficulties created by a psychometrically thin and conceptually weak articulation of "affectiveness" (l. 94), "meaningfulness" (l. 639) etc., the authors would do well to say something about the steps that might be taken in future efforts to provide psychometric substance to the construct they are trying to assess. References to Shklovsky and Miall are not enough (e.g., is "strikingness" the same as "affectiveness"? how would we know?). Very briefly, please say how this psychometric issue might be addressed in future studies.

**Review 1’s suggestions to further explain how, in future experiments, might refine our definition of ‘aesthetic pleasure’, which were, following Giora et al., perhaps overbroad, are much appreciated. We have, in response, added a final note to our Discussion section indicating the possibilities of using both Schindler et al.’s survey and Aesthetic Emotions Scale and Kuiken and Douglas’s Absorption-like States Questionnaire to clarify this definition and refine our questions for future experiments. See lines 747–765.**

Second, it is not clear that the authors have precisely coordinated their use of word2vec with the the DIFFERENTIATION that Katz and Al-Zary (2017) offer between (a) the distance between the vehicle and topic semantic neighborhoods and (b) the density of the separately considered vehicle and topic (l. 301). Is it possible to more precise about how these two aspects of metaphoric semantic structures are related to the procedures they rely on?

Reviewer #2: The authors have addressed the points I brought up in the last review. I think the manuscript looks excellent and will be very interesting to the PLoS ONE readership.

There is one small point of clarification about Katz and Al-Azary's (2017) Semantic Neighborhood Density (SND) measure. It is not exactly synonymous to semantic distance where two terms are compared, but rather, it is a measure of the average distance from a single term to its own neighbors. So, for a metaphor like "ski for office", SND doesn't actually measure the overlap between ski and office. Rather, it measures the semantic density of "ski" on its own and "office" on its own. If the sentence was "ski downhill", the SND for "ski" is still the exact same. It may indirectly get at overlap -- if "ski" and "office" both have high SND, then they may have more overlapping near neighbors, but SND on its own is not a measure of this. Just wanted to clarify this because the way it is written in the manuscript, it sounds like SND is a measure of the overlapping semantic neighbors between two terms.

They explain Semantic Neighborhood Density in a little more detail in this paper:

Al-Azary, H., McAuley, T., Buchanan, L., & Katz, A. N. (2019). Semantic processing of metaphor: A case-study of deep dyslexia. Journal of Neurolinguistics, 51, 297-308.

Thank you very much for the opportunity to review this work!

**We thank both the reviewers for their requests and suggestions for additional clarification on the potential relevance of semantic density (SND) to our study. We have removed our erroneous suggestion that our use of word2vec aligned with semantic density in our ‘Objective Measures’ subsection (l. 320–322 – formerly l. 301), as this was truly designed to measure distance between nouns and their interactive verbs within each condition, and between critical verbs between conditions.**

**A brief discussion of Al-Azary and Buchanan’s (2017) study of the influence of SND’s on novel metaphor comprehension is included in the introduction section (ll. 245–260). This replaces the less-appropriate discussion of Katz and Al-Azary (2017) (formerly l. 188). This article’s examination of SND and the bidirectionality of metaphors seems, upon reflection, to be less directly relevant than the Al-Azary and Buchanan’s (2017) examination of SND, concreteness, and metaphor comprehensibility.**

**A discussion of the possible influence of SND and difficulties of calculating and comparing the SND in our verb-metaphor stimulus set has been added to the Discussion section, lines 688–707.**
